# How Does the University Food Environment Impact Student Dietary Behaviors? A Systematic Review

**DOI:** 10.3389/fnut.2022.840818

**Published:** 2022-04-08

**Authors:** Xingbo Li, Andrea Braakhuis, Zengning Li, Rajshri Roy

**Affiliations:** ^1^The University of Auckland, Auckland, New Zealand; ^2^The First Hospital of Hebei Medical University, Shijiazhuang, China

**Keywords:** dietary behavior, food environment, university student, nutrition, diet quality

## Abstract

The food environment has been implicated in creating an obesogenic generation; and while previous research has focused on population-wide initiatives, the university population resides in a research gap. To explore detailed components of the university food environment and what shaped dietary behaviors, we retrieved literature from the following databases: ScienceDirect, Scopus, PubMed, Medline, and EMBASE. Eleven studies were identified for qualitative content analysis and study quality assessment identified most of the studies as of good quality (*n* 8) and some as of fair quality (*n* 3). The certainty of evidence remained at a low level for all (*n* 11) studies due to the cross-sectional, observational nature of studies. Three major themes emerged: (1) food environment, (2) student perceptions (SPs), and (3) dietary outcome (DO). In a university food environment, the taste of food was paramount for the food choices of students, followed by the availability of food and the price of food. When university students perceive and choose foods and beverages, they sometimes regard unhealthy foods and beverages as healthy options. The diet quality of university students is more susceptible to living arrangements and socioeconomic status, but higher salt, fat, and added sugar consumption generally resulted in poorer quality of diet on campus. Future research could use novel methods to explore a wider range and deeper level of students' dietary behavior determinants in university food environments.

**Systematic Review Registration:**

https://www.crd.york.ac.uk/prospero/, identifier: CRD42021283562.

## Introduction

Non-communicable diseases (NCDs) are statistically responsible for 71% of global deaths with unhealthy diet listed as one of the five major risks for NCDs (1). Global concerns have been raised regarding the public health issues of overweight and obesity where their prevalence has reached 38.9 and 13.1% in adults, respectively (2). Obesity has evolved beyond the point where it was viewed primarily as a behavioral outcome related to individual willpower but is now considered a multifactorial “disease” (3). Many individual factors including genes could affect obesity outcomes, yet cannot explain, to a satisfactory extent, why the obesity epidemic grows rapidly in recent decades (4). From environmental perspectives, multiple studies have attempted to answer why obesity has increased at a dramatic rate; but given the lack of systematic methodology and reliable longitudinal data, questions still remain (5–7). Although some data suggested a null association between the food environment and obesity, more recent evidence on adults showed substantial susceptibility to factors including convenience store proximity, restaurant density, and direct food availability (8–10). In an urban living environment, consumption of modern ultra-processed foods (11) and high energy dense foods (12) have accelerated the progression of this epidemic. Recent investigations report that consuming diets high in ultra-processed food causes excess energy intake, weight gain, and may exacerbate metabolic syndrome (13).

Much effort has focused on investigating the effect of the food environment on dietary behavior and health status in different settings (14–16). Besides choosing different food environment settings to investigate such relationships, population groups are also eligible options. While certain studies assumed earlier life stages are more influential on food choices of individuals than later stages in life (17–20), intervention work on children has found contrasting results. The effectiveness of food environment interventions in kindergarten and primary school settings was less than convincing (21, 22). On the other hand, adults have also been studied for their dietary behaviors under workplace food environment settings (23–25). Several studies have illustrated how interventions and modifcation to the food environment can lead to changes in the dietary behavior of adults in the community and workplace (26–28). University or college students are in the transition from adolescence to adulthood, but there were few studies that could provide evidence on how their dietary behaviors are influenced by their surrounding food environment.

Unlike secondary schoolers, young adults enrolled in tertiary educational institutions living away from home are no longer under strict family supervision for daily dietary intake. According to student accommodation surveys, the proportion of university or college students living at home with parents was as low as 10 to 16% (29, 30). Studies have concluded that university students are subject to rapid weight gain, especially in their first year of study (31–34). This population is particularly at high risk of developing unhealthy eating habits and subsequent health problems such as obesity and diabetes (35–38). The university food environment is composed of a relatively fixed variety of options and closer contact with individuals, especially when the food outlets are on campus. Despite many interventional studies (39–42), the relationship and confirmed relatedness between university food environment and student and staff dietary behavior have not been established well.

The current review aims to explore whether the university food environment influences university students' dietary behaviors, and to understand how university students perceive their food environment. In particular, we investigate evidence on how specific components of the university food environment impact food choices, dietary intake, eating behavior, and diet quality of students.

## Materials and Methods

The systematic review was conducted according to the PRISMA 2020 Guidelines (43), adapted to public health intervention outcomes.

### Eligibility Criteria

In order to select relevant articles, the following inclusion criteria were applied: randomized controlled trials (RCTs), pre- and postintervention studies, quasi-experimental studies, cross-sectional studies, and other non-experimental or pragmatic design studies; participants studied for their outcomes in a tertiary education setting; primary outcomes included measures or changes to dietary behavior; and studies targeted at students attending university/college. The inclusion criteria are fully detailed in Table 1. Exclusion criteria included studies that did not focus on food environment or DOs of participants; any physical activity interventions; and interventions conducted in settings other than universities/colleges.

**Table 1:** Criteria for selecting eligible articles for review.

**Study component**	**Inclusion criteria**
Study design	Randomized controlled trials (RCTs), pre- and postintervention studies, quasi-experimental studies, cross-sectional studies, and other non-experimental or pragmatic design studies.
Study characteristics	Full text written in English; published up to October 2021.
Population	University/College students; age was not specifically limited.
Study setting	The participants should have been studied in a university setting where their dietary behavior is affected by food environment of the university.
Outcomes	Main outcomes related to dietary behavior include food choices, eating habits, and food components; the comprehensive taxonomy applied to this review has been defined previously (44).

### Data Sources and Search Strategy

**Figure 1:**
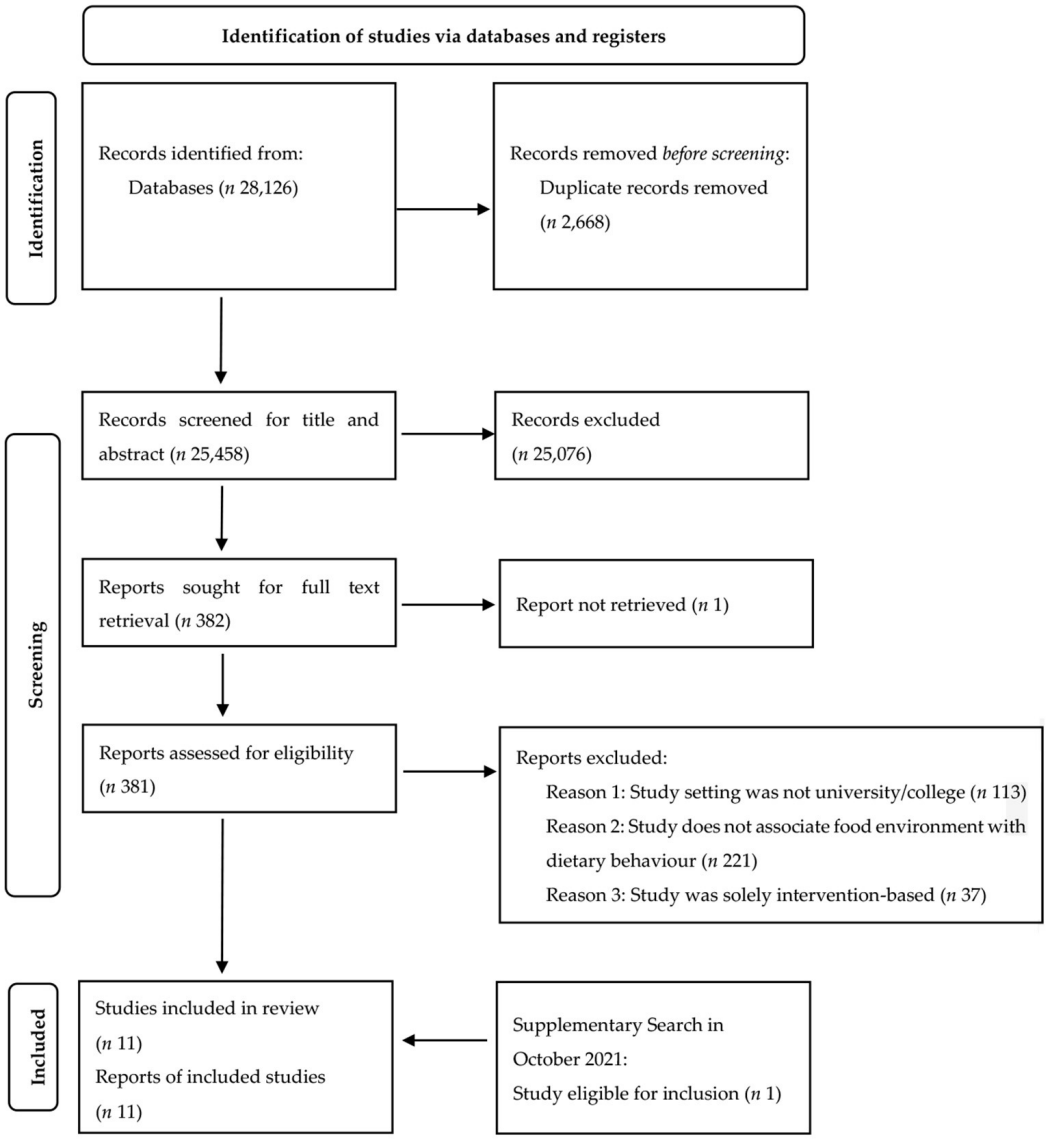
Flowchart of literature search and selection process.

Conducting and reporting this systematic review was based on the preferred reporting items for systematic reviews and meta-analyses (PRISMA) statement (43). The PRISMA flowchart for literature search and selection process as been provided in Figure 1. To retrieve literature on this topic, keyword search was conducted within seven databases closely relevant to nutrition and public health research: Web of Science, Scopus, ScienceDirect, PubMed, Cochrane Library, Medline, and EMBASE.

Basic logic for database search strings included four domains: (1) one domain defining the study as university setting; (2) one domain indicating food environment as the influential factor; and (3) two domains investigating dietary behavior as the outcome. Boolean operators and MeSH terms were incorporated into the search strategy when appropriate. Table 2 described the detailed search operations in all databases. Specifically, the year of publication was not restricted for result retrieval, but publication type has been recorded wherever applicable. The main body of the search was completed in November 2020. Upon removing duplicates, 28,126 records were imported for screening. A supplementary search was performed in October 2021, which identified one additional study that met the inclusion criteria.

**Table 2:** Keyword and search strategy used in each database.

**Database**	**Search string**	**Filters limitations**	**No. of results**
Web of science	(food environment) AND ((dietary behavior) OR diet OR intake OR consumption) AND (university OR college) AND nutrition	None	552
Scopus	(TITLE-ABS-KEY(food AND environment) AND TITLE-ABS-KEY(intake) AND TITLE-ABS-KEY(university) OR TITLE-ABS-KEY(diet) OR TITLE-ABS-KEY(consumption) OR TITLE-ABS-KEY(college))	None	8,314
ScienceDirect	(“food environment”) AND ((dietary behavior) OR diet OR intake OR consumption) AND (university OR college)	None	4,965
PubMed	(university OR college) AND (diet[MeSH Terms]) OR eating[MeSH Terms] AND (food environment)	None	16,838
Cochrane library	food environment	The search identified 15 review entries, but none met inclusion criteria.	
Medline	“food environment” and (universit* or college) and (intake or diet* or behavior or consum*)	None	64
EMBASE	“food environment” and (univer-sit* or college) and (intake or diet* or behavior or consum*)	None	100
		**Total**	**28,126**

### Selection Process

One reviewer was involved in title and abstract screening; entries that were book chapters or non-journal articles were excluded; study focus not related to food environment and/or dietary behavior were excluded; and setting other than university/college were excluded. The definition of a university food environment has been determined to be considering on-campus food venues, restaurants, café, vending machines, and food sources readily available to students who are physically attending university. One report screened contained only a poster abstract of the research conducted (45). A request for full text or further details regarding the research was sent to the corresponding email address without a reply for over 60 days and was thus excluded from the final review list. Two reviewers independently reviewed the proposed list (*n* 14) for review and reached a consensus on the final list (*n* 11). Out of the three studies not included, two were excluded because they were only concerning interventional outcomes rather than food environment measurements (46, 47) and one focused on discussing food environment intervention policies (48).

### Certainty of Evidence

The certainty of the evidence was assessed using the GRADE system. Certainty of evidence of cross-sectional observation studies was at most low-level certainty (49), as defined by the GRADE criteria. In this review, we also assessed the quality of each included study using the National Heart, Lung, and Blood Institute (NIH) quality assessment tool (50). Two reviewers independently used the NIH tool to assess the study quality and reached a consensus. No disagreements on final assessment outcomes were raised in the process of this review.

### Data Synthesis

Although all included studies were cross-sectional observational studies, significant heterogeneity in country settings, sampling design, and outcome measures meant that data could not be pooled for a meta-analysis. Most of the literature included mixed quantitative/qualitative research focusing on food fact questionnaires (FFQs, *n* 9) and qualitative focus group discussion (*n* 2). Results were analyzed using an interpretive content analysis approach where the researchers reviewed and coded the results. Thematic categories were generated and combined after each iteration and researchers were finally in consensus with major themes.

## Results

### Overview of Studies

Initial screening for title and subsequent abstract review identified 381 articles for further full-text examination. The exclusion of articles was decided with care since we wanted to extract as much evidence as possible on this understudied area. A final list (*n* 11) was reached after a thorough assessment of study contents: all were cross-sectional studies with survey design (*n* 9) and focus group discussion (*n* 2) methodology. Three studies were determined to be of a very-low level of certainty due to their lack of essential quality assurance measures within study design and conducting, other eight included studies remained as low-level evidence. Details of the assessment are summarized in Supplementary Table S1.

The characteristics of included studies and their primary contribution to this review have been tabulated in Table 3 and are briefly described below in the text, classified into three themes using content analysis based on what aspects of the food environment were investigated in the studies. The food environment theme included studies related to the examination of the unique food environment (FE) within tertiary education settings (*n* 5); studies (*n* 4) fell under the SPs of the university food environment theme; and DO theme-related studies (*n* 6) that looked at the exposure to such food environments and its impact on dietary behaviors of participants.

**Table 3:** Summary of included studies and the methods used.

**Citation**	**Research emphasis**	**Theme(s)**	**Relevancy**	**Nation**	**Methodology**	**Strengths**	**Limitations**	**Results**	**Summary of findings**	**Study quality**
Kourouniotis et al. (51)	**Variable**: importance of taste **Outcome**: association with diet quality	SP & DO	**Strong** Taste was discretely studied as a factor influencing dietary behavior among university students.	Australia	**Research design**: observational survey **Sampling design**: convenience **Samples**: 1,306 students with mean age 20.6 years	1. In-depth understanding of one food environment factor 2. Quantitatively evaluated the level of influence of taste	1. Dietary recall may not reflect actual food consumption 2. Taste remains difficult to intervene due to subjectiveness	Majority of participants (82%) regard taste as a “very important” determinant in their food choices. However, among them diet quality was poorer. Fruit and vegetable consumption were significantly lower, too.	Taste of food found within universities is an important factor for student dietary behavior.	Good
Kremmyda et al. (52)	**Variable**: home/university food environment **Outcome**: dietary pattern changes	FE & DO	**Strong** This study presented evidence that the university food environment could change student dietary behavior.	UK	**Research design**: observational survey **Sampling design**: previous cohort recruitment **Samples**: 55 + 43 + 37 = 135 students	Compares home and university food environment to explore which can cause a change in dietary behavior	1. Study population may have been more health conscious 2. No collection of qualitative data	Students who continued to live at home maintained their dietary patterns after enrolling at university. Those living away from home consumed significantly less meat, cheese, and fresh fruits.	University food environment and dietary behavior of surrounding students may result in changes in existing dietary pattern of newly arrived students.	Good
Van Den Bogerd et al. (53)	**Variable**: lifestyle characteristics **Outcome**: fruit and vegetable (F&V) consumption	FE & DO	**Moderate** This study associated lifestyle factors with fruit and vegetable consumption.	Netherlands	**Research design**: observational survey **Sampling design**: convenience **Samples**: 717 students aged 22 years on average	Besides quantitative analysis, this study also collected qualitative opinion on interventions	1. Only one aspect of university dietary behavior included 2. Response rate was low, suggesting potential bias	Participants agreed that the university food environment contains enough healthy foods (60 %) and F&V (65 %), but also prefer more affordable F&V.	Healthy food options were available on campus, but will of students to purchase them may depend on how university vendors sell them.	Fair
Block et al. (54)	**Variable**: beverage choice factors **Outcome**: beverage purchasing behavior	SP	**Strong** This study explored how beverage could influence student purchasing behavior.	US	**Research design**: focus groups **Sampling design**: convenience **Samples**: 90 students aged 19 years on average	1. Diverse groups reached a consensus 2. Participants expressed diverse qualitative responses	1. Relatively small sample size 2. Manifested information were not interpreted and discussed	Taste was the most important factor followed by price. Participants showed little interest in health and nutritional value. Juices, regardless of their actual ingredient, are considered to be healthy.	Taste remains the most important factor among students when selecting for beverages.	Fair
Roy et al. (55)	**Variable**: food outlet healthiness score **Outcome**: purchasing behavior	FE & SP	**Strong** This study systematically audited university food environment. It examined how student dietary behavior can be affected by various factors.	New Zealand	**Research design**: observational survey **Sampling design**: convenience **Samples**: 1,954 on-campus student/staff under 25 years	1. Robust and replicable study 2. Large sample size	1. Alcohol-related food contents not examined 2. Excluded food outlets outside campus boundaries	Median food environment-quality index was 79 out of 199. Six food outlets were categorized as healthy and two as unhealthy; the rest were intermediate. Overall, healthy items were less available, accessible, and promoted and cost more than unhealthy items.	The university needs to improve the availability and variety of healthy foods on campus. Value for money is another factor that influences healthier food choices.	Good
Hebden et al. (56)	**Variable**: food choice factors **Outcome**: importance of each factor	SP	**Strong** This study does not investigate environment but the underlying factors and how participant demographics can influence.	Australia	**Research design**: observational survey **Sampling design**: convenience **Samples**: 112 students aged 19-24 years	1. Considered socioeconomic status 2. Used standard physical activity assessment tool	Study population shows significantly healthier anthropometric measures	Weight control diet is preferred in students with higher waist circumference. Consumption of healthy foods and foods high in nutrition value were reported by physically active individuals.	Taste was important, but the level of influence may depend on individual's body shape and physical activity level.	Fair
Sogari et al. (57)	**Variable**: factors of the food environment **Outcome**: barrier or enabler to healthy dietary behavior	SP	**Strong** This study summarized factors from socioeconomical to individual aspects.	United States	**Research design**: focus groups **Sampling design**: convenience **Samples**: 35 students aged 19–25 years	1. Comprehensive coverage of potential factors 2. Used validated analysis software	1. Small sample size 2. Entirely based on subjective quantitative responses	Barriers to healthy eating: tight time schedule, highly accessible unhealthy foods, and costly healthy food options. Facilitators for healthy eating: nutrition knowledge education, meal planning and preparation, and regular physical activity.	Healthy foods appear to cost more time than easily accessed junk foods, restricting students from choosing healthier options.	Good
Fonseca et al. (58)	**Variable**: food choices **Outcome**: pattern of food consumption	DO	**Moderate** This study focused on a snapshot of choices of students rather than a relationship between food choices and the university food environment.	Brazil	**Research design**: observational survey **Sampling design**: convenience **Samples**: 685 students aged 19–24 years	Connects food environment and dietary behaviors to a well-established dietary pattern (DP) field	The study focused more on the relationship among different dietary patterns rather than food environment.	Three DPs were extracted. Students consuming meals on campus showed at least adherence to one DP that is different from their home food choices. Socioeconomic status affected the DPs followed in some participants.	Students choose set combinations of foods that may result in routine consumption of unhealthy foods.	Good
Pelletier and Laska (59)	**Variable**: food purchase and diet quality **Outcome**: healthy eating behaviors	FE & DO	**Moderate** Food purchasing on campus has been associated with less healthy dietary outcomes.	US	**Research design**: observational survey **Sampling design**: convenience **Samples**: 1,059 students with mean age of 22 years	Large sample size, diverse sample demographics	University food and home-prepared food were qualitatively assessed on different scales.	On campus food purchase was associated with less frequent breakfast consumption and higher fat and added sugar intake. Home-brought foods are associated with healthier overall dietary pattern.	Students may purchase foods from university environment but the foods provided on campus were found to be less healthy.	Good
Roy et al. (60)	**Variable**: food purchase **Outcome**: dietary quality factors	DO	**Strong** This study showed how food purchasing behavior could be associated with food group and nutrient intake.	Australia	**Research design**: observational survey **Sampling design**: convenience **Samples**: 103 university students aged 19–24 years	1. Assessment of dietary quality was completed via validated tools 2. Participants used 5-d WFR, which produced higher quality data	1. Sample may have been higher in healthy eating consciousness and socioeconomic status due to data collection methods	Frequent on-campus purchases lead to a significant decrease in diet quality; body mass index and waist circumference decreased as the HEIFA score increased.	Frequent on-campus food purchasing suggested poor diet quality. Food nutrition quality on campus needs improvement.	Good
Martinez-Perez et al. (61)	**Variable**: food outlet healthiness score and NOVA food processing level **Outcome**: student and staff purchasing behavior	FE	**Strong** This study measured the healthiness and processing level of foods available on campus. It also investigated perceptions of students of their food environment.	Norway	**Research design**: observational survey **Sampling design**: convenience **Samples**: food environment audit: 12 outlets; purchase behavior: 129 participants.	1. Robust and replicable study 2. Surveys opinion of participants on food environment 3. Included food processing level	1. Excluded food outlets outside campus boundaries. 2. Very similar to a previous study (52) with similar conclusions.	Food environment: 39·8% of the products were “unhealthy” and 85·9% were “ultra-processed.” Food purchase behavior: participants reported on taste, cost, and convenience as determinants for dietary behaviors.	Two prevalent suggestions: healthy food at a lower cost and more variety of foods.	Good

*FE, food environment; SP, student perception; DO, dietary outcome*.

### The University “Food Environment”

Five cross-sectional survey-based studies commented on the university food environment that students were exposed to. Two studies performed detailed food environment audits to assess food outlets and vending machines on the products sold, while the remaining three studies employed relatively more subjective responses or collective descriptions for food environment evaluation. However, audit results were contradicting: Martinez-Perez et al. (61) identified unhealthy food and beverage options including sweet snacks and sugar-sweetened drinks as significant components of solid food (58.5 %) and total drinks (23.5%), respectively. However, Roy et al. (55) observed a higher number of “healthy” outlets (17.8%) suggesting that healthy food options were available on campus and were higher in density than less healthy ones (3.6%). In the same study, however, participants reported less healthy food and beverage purchases on campus. They indicated poor value for money and insufficient healthy food options as the main reasons. The conflict between SP of healthy food availability and author audit outcomes implied a mismatch in their ability to accurately identify foods as healthy or unhealthy.

Van den Bogerd et al. (53) collected information from student responses on dietary intake and food availability in the university food environment particularly concerning fruit and vegetable intake. Approximately a quarter of the students met fruit intake recommendations (27.9%) compared to a very low proportion for adherence to the vegetable guidelines (6.8%). Similar to Roy et al. (55), students commented on the low availability of desirable food products. They expressed opinions on increasing affordable fruit and vegetable options. But the study failed to coordinate participant response with the actual university fruit and vegetable availability.

A considerable proportion of students' dietary intake took place outside the campus wall in non-campus nearby stores, especially for those living off-campus (59). Compared to students living with family, participants in this study exhibited higher fast-food consumption and less healthy dietary patterns (DPs). However, the healthiness index of products available at food outlets has not been assessed in this study, and the authors did not synthesize whether the university food environment promoted unhealthy food consumption through price, availability, or accessibility.

In addition to the food environment created by university food outlets and what was available at the outlets, cultural norms and peer influences were found to change DPs of university students, particularly for those from a different background (52). In this study, Greek participants were recruited to provide dietary behavior information in four groups: studying in/out of Greece and away/not away from home. Instead of enlisting single components of the university food environment, the authors noted a “Western” style of foods and beverages available to students who originally adhered to a Mediterranean DP. Quick acculturation to the local style of food consumption suggested that students exposed to altered food environments could demonstrate dietary behavior localization along with attempts to integrate into the local population.

### “Student Perceptions” of Factors Influencing Dietary Behaviors

Out of the four studies that focused on the perceptions of students on their food environment, two used focus groups whereas the other two used cross-sectional survey-based methods. All studies concluded that taste was the most important factor when they made food and drink choices. Block et al. (54) reported how college students perceived and consumed sugar-sweetened beverages by analyzing results of 12 focus group discussions with an average participant age of 19 years, where some participants even commented that they “can not resist” the taste of certain drinks. Hebden et al. (56) administered online surveys on students aged 18–24 years and concluded that taste was rated as the most important factor based on an integer scoring system from 0 to 3. Participants recruited from nutrition class rated “quality” as an important factor but still ranked taste as the most influencing factor for their dietary choices (51).

Following taste, three factors were frequently placed on responses of the students: value for money, convenience/availability, and nutrition value. Price could be placed as the second most important influencing factor but the dominance of taste over price was clearly identified and a lower price only mattered if taste was not compromised for less cost (54). Nutritional value emerged as one of the factors; however, mainly due to the needs of participants for weight control and physical activity routine (53, 56).

In the included studies, gender difference also appeared to influence factors defining dietary behavior of university students. Among participants of Hebden et al. (56), higher physical activity levels have been linked to decreased importance on taste as a driver of food selection, particularly in regularly exercising females. In the studies from Kourouniotis (51) and Roy et al. (55), however, female participants were found to pay more attention to how palatable foods were when they select for foods possibly because these two studies did not measure physical activity levels. Female participants demonstrated more awareness especially for accessibility, appearance of foods, and weight control properties; males, on the other hand, showed significant preference over foods that keeps them awake or alert, are familiar, and helps cope with stress (55).

### “Dietary Outcome” and Quality in a University Food Environment

Six cross-sectional survey-based studies reported on the outcomes of dietary behaviors of university students. Two of the six studies introduced DPs as an outcome measure to assess student dietary intake. Fonseca et al. (58) provided novel insights on DP of students rather than individual dietary components by conducting self-administered questionnaires in a Brazilian university. By statistically analyzing consumption patterns of individual foods and drinks, the authors concluded three DPs for each meal: breakfast, lunch, and dinner. In the university food environment, the studied population demonstrated preferences to consume foods that contain higher levels of simple sugars at breakfast and fried foods and processed juice when consuming lunch at university. However, higher adherence to a healthier DP was observed among students who consumed dinner at university, especially for students with lower socioeconomic status.

In another study employing DPs to measure student DOs, Kremmyda et al. (52) compared Greek college students who continued to live at home (*n* 43), lived away from home but in Greece (*n* 37), and lived away from home in UK (*n* 55). Students who remained in Greece did not show significant alterations in their dietary behaviors, whereas those who moved to the UK changed their routine diets. Although the authors attributed such observation to a general difference between the Northern European dietary environment and the Mediterranean one, no in-depth analysis of the food environment differences was conducted within this study.

Van den Bogerd et al. (53) measured fruit and vegetable intake of university students to find that international, independently living, male, and moderate-to-excessive alcohol drinkers were more frequently not consuming fruit and vegetables. Similarly, vegetable and fruit consumption was reduced in Greek students who moved to the UK, while French fries and savory snack consumption increased (52). Less fruit and vegetables were also reported by Kourouniotis et al. (51) in a larger studied population especially among participants who were concerned more about taste.

In addition to fruit and vegetable intake, Kourouniotis et al. (51) found that when university students rated taste as the most important factor influencing their food choices, they often reported higher likelihood to consume foods high in fat, salt, and sugar; and less consideration for healthy dietary options such as avoiding adding salt to cooking and adding sugar to tea or coffee. In an unadjusted analysis, Pelletier and Laska (59) associated frequent purchasing campus area food/beverages with a DP of higher consumption of fat and added sugars and lower consumption of dairy. Similarly, eating fast food >3 times per week was associated with higher consumption of fat and added sugars. Students who frequently brought food from home exhibited lower fat consumption and added sugars and higher consumption of dairy, fruits and vegetables, calcium, and fiber.

In two studies concerning the frequency of food purchase on campus, more than half participants reported consuming five or more foods and/or beverages on campus per week regardless of gender (58, 60). When the authors stratify food and beverage purchase frequency and compare that with participant dietary quality score, more on-campus purchases led to poorer diet quality (51, 60).

## Discussion

Overall, the university food environment impacts dietary behavior of students mostly in unfavorable ways to ultimately cultivate an unhealthy eating style (57–59). Student demographics, body shape desire, and social dietary interactions added to the influence of university food environment (52, 56, 58). The impact from university food environment originates from its components and is then amplified with perceptions of students about the food environment: taste, price, and accessibility (55). The gap between understandings of participants of what “healthy food” is and the objective assessment outcomes of its healthiness may have contributed to this effect and disguised underlying needs to modify the university food environment (62).

The university food environment has been characterized by low availability of healthy foods and higher cost compared to unhealthy options (63–65). When students perceived foods provided on campus, the close availability of junk foods and costly fruits and vegetable options prevented them from reaching for healthier dietary choices (57, 66). Although food environment audits evaluated certain critical aspects, some potentially powerful factors were overseen and few have assessed important non-geographic dimensions of availability (53, 55). For example, food outlets within the same premise could expose healthy or unhealthy product differently to customers and result in distinct purchase behaviors (67).

When university students, or young adults who were primarily 18 to 30 years old, reported their purchase determinants within the university food environment, they considered taste as the paramount factor for dietary options, followed by cost and availability (51, 54, 55). Factors that are less frequently mentioned or placed at lower ranking included tight schedules and convenience of consumption (54, 57). Similar factors were found to impact dietary choices among middle-aged adult population (68), in workplace (69), or school settings (70). However, based on the low level of evidence certainty, we explored factors influencing dietary behaviors in settings other than university and among other age groups. Children between 6 and 12 years subjectively chose, within their knowledge, foods with low nutritional value from school canteens (71, 72) whereas socioeconomic factors played a major role in food choices when individuals were aged 30 years (73). University student population are transforming from low- to high-nutrition value preferences but limited by costly healthy food options (74, 75). Before cost efficiency dominates dietary behavior in later adulthood, university students are susceptible to respond to interventions and adopt healthier dietary styles before they lose interest in considering nutritional value of their food and drinks.

Two studies concluded that university students experience stress from many aspects and the overall effect is overeating and consuming high-calorie foods (56, 57). However, the studies only included a small number of participants (*n* 112 and 35, respectively) and employed methods that were relatively weak in reliability, implementing the risk of participant expectation bias (76). Mindful eating has been related to students trying to maintain desired body mass index and cope with their physical activity levels (77). Studies have focused on associating levels of stress, anxiety, and depression with eating behaviors based on existing scales (78, 79), but failed to synthesize why students experienced stress that can be intervened from a food environment perspective.

Kremmyda et al. (52) described international student dietary behavior acculturation to local students; and friendship network has also been identified as an important influencing factor in child or adolescent population (80). However, a review on dietary behaviors of the elderly concluded contradicting conclusions on whether living arrangements had an impact on diet of the participants (81). For university students, results from one study suggested little or no effect of influence of friends on their dietary behavior (82). Because the nature of social interactions differs dramatically for universities, further evidence would be needed to conclude the effect of sociocultural factors.

Although interventional studies were not the focus of the current review, they added insights to the research question. Young adults in universities have been found to consume foods at the portion sizes they were served with and increase their consumption as servings become larger (83). Rolls et al. (84) commented on the positive effect of synergistically reducing portion size and food–energy density to promote less total energy intake among university students. Means of food acquisition for university students have also increased, especially when students could access more affordable delivery options (85, 86) despite them being the least healthy type of food acquisition compared to the university dining hall, sit-down restaurants, and fast-food options (87). However, studies in workplace settings identified portion size interventions as non-effective in reducing total food intake in adults (88). In our review, evidence is lacking on how serving sizes in a university food environment could influence the dietary behaviors of students.

## Strengths and Limitations

This review features several strengths that contributed to the robustness of evidence synthesis. First, the search strategy is comprehensive and does not apply any filters or year of publication limits. The records retrieved covered literature published in more than 90 years from 1930 to 2022. The reviewers generously performed title and abstract screening to allow as much evidence to be examined as possible. For a population of mixed-method studies, we employed the NIH Study Quality Assessment Tool to specifically focus on assessing the qualitative contents of the research while identifying advantages and flaws in quantitative designs. The research team abnegated a meta-analysis approach and switched to an interpretive qualitative content analysis approach to synthesize evidence. This substantially mitigated inconsistencies in study design and data collected across included studies and allowed deeper insights to be drawn from the pool of results, which would otherwise be discarded for meta-analysis.

However, some limitations should be noted. Study heterogeneity has been found in studies on food environment, particularly when it was associated with dietary behavior under diverse university settings. Among included studies, only one study claimed to have collected longitudinal cohort data but only with two timepoints (52), while others employed either a cross-sectional survey (*n* 8) or focus group (*n* 2). All included studies (*n* 11) employed convenience sampling methods, which could potentially reduce the representativeness of the sample and hence lower reliability. The association between university food environment and student dietary behavior still lacked high-quality longitudinal study to provide stronger support.

Confounding was lower-ranked on the priority list of consideration among studies and may or may not be evident enough to confirm the association between university food environment and dietary behavior of the student. The effect could have been hindered among the vast variety of factors influencing dietary behavior in a real-world university setting (89). Heterogeneity in population characteristics and lacking comparable parameters limited the generalizability of overall findings. Cross-sectional studies bear a nature of low certainty of evidence, hence the findings may or may not reflect the true underlying motives for dietary behaviors of university students.

## Implications for Future Research

Subjective perceptions of the food environment from students could be as important as or even more important than the options they are exposed to but unfortunately was often overseen in some studies attempting to answer the question of what shaped their dietary behavior. Non-geographical features affecting the accessibility to food options of students were one of the factors unexplored in current methods (90). Determining factors that influence the dietary intake of university students require protocols beyond a textual questionnaire to elicit the opinions of participants (91).

Photographic and audio/video sampling from participants, especially young adults, could be a valuable tool for researchers to synthesize themes that participants might not even be aware of. Photographic food record assessment has been employed to measure dietary intake, although this method was found inaccurate among populations with distinct characteristics (92). The validity and applicability have been established in settings such as adults eating ad libitum (93, 94), hospitalized patient diets (95), in school cafeterias (96), and in collective dining food environments (97, 98), and could be considered as a viable university food environment research methodology.

Food environment factors promoting stress eating and unhealthy food options more accessible to susceptible stress eaters could be the future interest of investigation. Further research should focus on determining the potential for long-term food environment interventions and collect reliable longitudinal data to assess whether modifications to the university food environment impact student dietary behavior. The university staff population was understudied for their dietary behavior within the university food environment. Comparison and contrast of staff and student population who were significantly different in demographic characteristics could indicate the critical relationship between the food environment and how dietary behavior is affected.

Controversial opinions on the impact of individual behavioral factors on dietary behavior among university students indicate further research to understand more on this aspect. Morin et al. commented on factors that could shape the dietary behaviors of individuals including fear of social isolation and altruistic motives such as sustainability awareness (99). In the reviewed studies, such social factors were not investigated because it would require assessment methods beyond cross-sectional questionnaire surveys. Collaborating descriptive and quantitative audits of the food environment with longitudinal dietary behavior data could potentially help successfully understand the determinants of the dietary behavior of university students.

## Conclusions

Significant factors determining food choices and eating habits of university students include taste, value for money, and accessibility of foods and beverages. Hence, interventions targeting the availability, accessibility, and cost of these unhealthy and healthy foods could be influential for obesity control among university students, particularly the first-years. Current results rarely report on sociocultural factors influencing the dietary behavior of university students. Future research is warranted on the collection of longitudinal data with revised methods to allow participants to demonstrate a more comprehensive picture of how the university food environment has shaped their dietary behavior.

## Data Availability Statement

The original contributions presented in the study are included in the article and Supplementary Material, further inquiries can be directed to the corresponding authors.

## Author Contributions

XL and RR: conceptualization, methodology, and formal analysis. XL, RR, AB, and ZL validation. XL: writing the original draft preparation and visualization. RR, AB, and ZL: writing, reviewing, and editing and supervision. RR and ZL: project administration. All authors have read and agreed to the published version of the manuscript.

## Funding

This study was funded by Internet Plus Clinical Nutrition Teaching Modes Innovation of Hebei Medical University, China [grant number: 2020YBZD-7] and Internet Plus Clinical Nutrition Teaching Modes Innovation of Hebei Education Department, China [grant number: 2020GJJG122].

## Conflict of Interest

The authors declare that the research was conducted in the absence of any commercial or financial relationships that could be construed as a potential conflict of interest.

## Publisher's Note

All claims expressed in this article are solely those of the authors and do not necessarily represent those of their affiliated organizations, or those of the publisher, the editors and the reviewers. Any product that may be evaluated in this article, or claim that may be made by its manufacturer, is not guaranteed or endorsed by the publisher.
